# Daily automated feedback enhances self-regulated learning: a longitudinal randomized field experiment

**DOI:** 10.3389/fpsyg.2023.1125873

**Published:** 2023-05-18

**Authors:** Henrik Bellhäuser, Charlotte Dignath, Maria Theobald

**Affiliations:** ^1^Department of Psychology, Faculty 02: Social Sciences, Media, and Sports, Johannes Gutenberg-University Mainz, Mainz, Germany; ^2^Center for Research on Education and School Development, TU Dortmund University, Dortmund, Germany; ^3^Individualised Interventions, Education and Human Development, DIPF Leibniz Institute for Research and Information in Education, Frankfurt, Germany

**Keywords:** self-regulated learning, individual feedback, learning diaries, ambulatory assessment, multilevel analysis

## Abstract

The goal of the present study was to investigate the effects of automatically generated, adaptive feedback on daily self-regulated learning (SRL) in an experimental field study. University students reported their application of SRL strategies in the morning and in the evening over the course of 36 days using electronic learning diaries. Students were randomly assigned to the experimental group with feedback (LDF, *n* = 98) or the control group without feedback (LD, *n* = 96). Based on their self-reports, students in group LDF received daily written feedback regarding their satisfaction with the study day, adherence to time schedule, procrastination, and effort. This feedback either reinforced students in their study approach (confirmative feedback), encompassed information on learning outcomes or processes (informative feedback), or included feed forward on how to improve learning processes (transformative feedback). Multilevel analysis of daily process data revealed better average goal setting, planning and adherence to time schedule, as well as higher self-efficacy, and satisfaction with the study day in group LDF compared to group LD. Motivation, procrastination and effort were not affected by feedback. In contrast to the process measures, pre-post comparisons of students’ self-reported general use of SRL strategies (trait measures) did not reveal any effects of feedback on SRL. Further explorative analyses investigated the effects of confirmative, informative, and transformative feedback on next day’s learning behavior, showing that confirmative and transformative feedback had stronger effects on students’ satisfaction and procrastination than informative feedback. Transformative feedback, which included specific strategies for moving forward, was effective in improving time management. Results provide theoretical insight into the interplay of feedback and SRL and offer practical implications regarding the design of feedback in a learning context.

## 1. Introduction

Self-regulated learning (SRL) describes the activities that a student performs in order to plan, monitor and regulate cognition, motivation, and behavior to achieve self-set goals (Zimmerman, 2002). SRL is a key competence associated with study success at all educational levels (Dent and Koenka, 2016; Theobald, 2021) and it lays the foundation for lifelong learning (Richardson et al., 2012; Schneider and Preckel, 2017). Since SRL is an iterative, cyclical process, learning activities such as study sessions are interconnected via internal feedback loops: The outcome of one learning activity (e.g., satisfaction) impacts the following learning activity (e.g., increased motivation) (Butler and Winne, 1995; Thurlings et al., 2013). External feedback that provides learners with evaluative information about their progress can enhance SRL by supporting monitoring and reflection (Butler and Winne, 1995). Hence, feedback constitutes a powerful, corrective tool to foster learning outcomes (Hattie and Timperley, 2007; Wisniewski et al., 2020).

Technological advancements allow for feedback to be generated automatically (Cavalcanti et al., 2021), thus providing an opportunity for cost-efficient large-scale interventions in educational settings. However, despite long research traditions for SRL and feedback (Butler and Winne, 1995; Nicol and Macfarlane-Dick, 2006; Panadero et al., 2018), this possibility is not investigated intensively in empirical research thus far—particularly not in the context of daily feedback through learning diaries.

We want to bridge this gap by analyzing the effect of individual feedback on SRL using a randomized control trial in a field study with fine-grained daily process measures of SRL. From a theoretical point of view, this study provides deeper insights into how – and more specifically which type of – feedback affects the SRL process. Further, results can inform practitioners on the linkage of two important areas of learning and instruction in order to support SRL among learners.

### 1.1. Literature review

#### 1.1.1. Self-regulated learning as a recurring process

**Figure 1:**
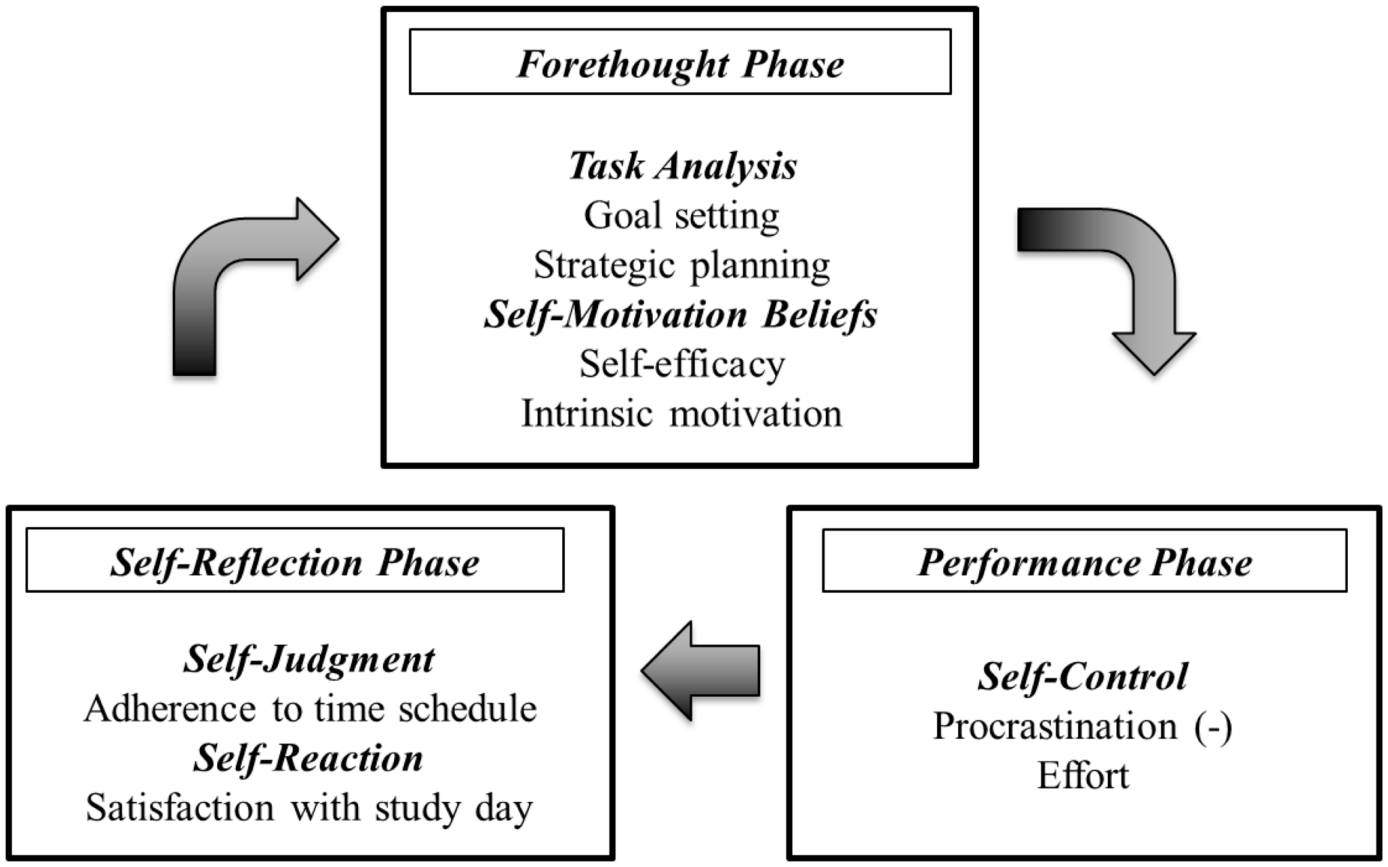
Adapted process model of SRL (Zimmerman, 2002).

SRL constitutes a multidimensional construct that encompasses cognitive, metacognitive and volitional strategies that students apply in order to attain self-set goals (Boekaerts, 1999). According to the process model of SRL (Zimmerman, 2002), each study session is divided into three phases: a forethought phase, a performance phase, and a self-reflection phase. An adapted version of Zimmerman’s process model builds the theoretical foundation of the current study, in which we focus on the variables presented in Figure 1.

In the forethought phase, learners set goals and make plans about how to proceed, whereby motivational states such as self-efficacy and intrinsic motivation affect how students approach their learning. Self-efficacy refers to learners’ judgments of their capabilities to organize courses of action to attain their study-related goals (Bandura, 1986). Research has repeatedly shown that self-efficacy is correlated with academic performance (Honicke and Broadbent, 2016). Intrinsic motivation “…refers to doing something because it is inherently interesting or enjoyable” (Ryan and Deci, 2000, p. 55) and is closely related to academic performance (Richardson et al., 2012).

During the performance phase, learners may choose cognitive strategies (such as organization or elaboration strategies) that support task execution. Meanwhile, they have to monitor their learning by observing whether they are still on track in order to perform the task (metacognitive strategies). Moreover, the application of volitional, self-control strategies supports the regulation of effort and ensures a continual, active engagement with the learning material. A lack of self-control strategies can result in academic procrastination, which refers to a “voluntary delay of an intended course of study-related action despite expecting to be worse off for the delay” (Steel and Klingsieck, 2016, p. 37). Procrastination is associated with lower academic performance, but can be reduced through interventions (van Eerde and Klingsieck, 2018).

In the self-reflection phase, learners evaluate their learning outcomes, i.e., whether they achieved their self-set goals (Winne and Hadwin, 1998). Thus, SRL is an active and constructive process whereby ongoing comparisons between the current and desired learning outcomes stimulate reflective processes. As SRL is a cyclic process, study sessions are interrelated via internal feedback loops (Thurlings et al., 2013). For instance, based on the learning outcomes, a learner could decide to change goals or learning strategies (Winne and Hadwin, 1998). Consequently, studying on one day depends on the internal feedback derived from the previous day, which in turn is likely to influence the forethought phase of next day’s learning process and the decisions that are taken such as goal setting and planning (Butler and Winne, 1995). Ideally, learners use the ongoing internal feedback loops to self-regulate their study activities which, in turn, depends on learners’ ability and willingness to monitor their studying (Narciss, 2008).

#### 1.1.2. Measuring and fostering SRL in daily life

Researchers frequently relied on retrospective self-report questionnaires to measure SRL (Roth et al., 2016). Yet, retrospective self-reports lack situation specificity (Winne et al., 2002) and not necessarily reflect a reliable and valid measure of students’ actual behavior in a given situation. Further, self-report questionnaires conceptualize SRL as a trait (Panadero et al., 2016b), whereas process models emphasize that SRL should rather be viewed as a state that varies over time depending on the learning context (Zimmerman, 2002). Hence, more fine-grained measures are needed to capture the dynamic adaptations in SRL strategies that occur during the learning process. Think aloud protocols, observations, or log-file analysis constitute valuable alternatives to retrospective questionnaires (Winne and Perry, 2000; Greene et al., 2011). However, these methods are time-and cost-intensive since raw materials need to be coded according to a coding scheme (Veenman, 2011) and therefore cannot by applied on a daily basis over an extended period of time. In contrast, diary methods capture “live as it is lived” (Bolger et al., 2003; Schmitz et al., 2011) and allow investigating individual differences in situative SRL over time.

Learning diaries, also sometimes called reflection protocols, learning-logs, or learning journals, typically contain open questions and closed Likert-type items, which cover the whole self-regulation cycle. Learners self-report their application of SRL strategies before and after a learning session, which complies with the dynamic nature of SRL and reduces the biases of retrospective questionnaires (Bolger et al., 2003; Klug et al., 2011). Variables of the forethought phase are measured before studying, whereas performance and self-reflection variables are assessed after each study session (Klug et al., 2011; Liborius et al., 2019; Bellhäuser et al., 2021). Thereby, learning diaries serve to measure SRL as a state in an ecologically valid setting since learners complete them in their natural learning environment (Schmitz and Wiese, 2006). Further, learning diaries can be used economically when the analyses focus on close-ended items that do not require extensive coding procedures.

However, measuring SRL with learning diaries is linked inseparably to reactivity effects: By prompting students to self-monitor their learning behavior, reflection processes are triggered that can lead to increased SRL behavior (Panadero et al., 2016b). Further, they can function as study reminders, stimulate reflection and enhance students’ awareness on the interrelatedness of different SRL components (Schmitz and Perels, 2011). While learning diaries seem to be a useful tool to foster SRL in school children (Glaser and Brunstein, 2007; Perels et al., 2009), findings on the effectiveness of learning diaries in the university context are inconsistent. Some studies revealed an increase in some SRL facets through diary keeping (Dignath et al., 2015), others did not find any effects on achievement (Bellhäuser et al., 2016), or even a negative effect on students’ intrinsic motivation (Dörrenbächer and Perels, 2016). Researchers suggested that learning diaries are only effective when integrated within a comprehensive SRL framework (Zimmerman and Paulsen, 1995) or when combined with a SRL training (Fabriz et al., 2014; Bellhäuser et al., 2016; Dörrenbächer and Perels, 2016). Otherwise, students might become frustrated since they do not know how to change their study behavior and lose their motivation (Panadero et al., 2016b).

External feedback that guides learners towards successful task completion constitutes one possibility to facilitate the transfer from reflection to actual behavioral change (Narciss, 2008; Shute, 2008). Receiving positive feedback on goal achievement should thereby also enhance learners’ motivation. For instance, repeated feedback on goal achievement should promote learners’ self-efficacy to achieve their goals (Bandura, 1986). In addition, feedback on goal achievement fosters perceived competence, which enhances students’ intrinsic motivation (Ryan and Deci, 2000).

However, providing external feedback takes time and effort and is therefore oftentimes not feasible for instructors when confronted with large groups of students. Technological advancements in recent years offer the possibility to generate automatic, adaptive feedback in a cost-efficient way. Yet, to date, research on the effects of automatically generated, adaptive feedback on daily reported SRL is largely missing.

#### 1.1.3. The interplay between SRL and feedback

Narciss (2008, p. 127) defines feedback as “all post-response information that is provided to a learner to inform the learner on his or her actual state of learning or performance.” Feedback is derived from internal sources of information (e.g., a learner self-monitors task performance or goal progress) or external sources of information (e.g., teachers, peers, or a computer) in order to reduce the gap between the actual level of performance and the desired goal (Evans, 2013). According to Hattie and Timperley (2007), effective feedback^1^ provides information on goals (*feed up*), current performance related to goals (*feed back*), and specific suggestions (*feed forward*) about how to close the gap between current performance and goals (see Shute, 2008, for a review on the design of effective feedback). Further, feedback can address four different levels (Hattie and Timperley, 2007):

First, outcome feedback includes information on task accomplishment, e.g., a student receives the correct solution to a task (Narciss, 2008). Outcome feedback, e.g., knowledge on results, is rather unspecific and provides little guidance on how to self-regulate learning (Butler and Winne, 1995) because it lacks the *feed forward* on how to proceed. Nonetheless, Vollmeyer and Rheinberg (2005) showed that students who expected to receive outcome feedback used better SRL strategies even before the feedback had actually been presented to them. Announcing feedback might cause learners to work more carefully as they expect an evaluation of their outcomes.

Second, process feedback draws the learners’ attention towards the relationship between the use of a specific learning strategy and their performance in order to induce deeper learning (Balzer et al., 1989). Process feedback typically includes *feed forward*, i.e., it encompasses strategic hints on how to proceed to overcome obstacles and to apply more efficient learning strategies (Shute, 2008). Hence, process feedback can be viewed as part of scaffolded instruction or as tutoring feedback (Nicol and Macfarlane-Dick, 2006; Narciss, 2008; Perry et al., 2008) that facilitates students’ monitoring and reflection, and thereby fosters the development of self-regulated learning strategies, which makes the process level a particularly good target for interventions.

Third, learners generate internal self-regulation feedback in addition to the abovementioned external feedback sources. This type of feedback is generated through self-monitoring of task engagement and performance, providing continual internal feedback on motivation, understanding, and goal progress (Butler and Winne, 1995). Internal self-regulation feedback is an important aspect of self-regulated learning, as it allows learners to adjust their learning strategies based on their self-reflection and self-assessment. By consciously engaging in reflective processes and modifying their SRL strategies accordingly, learners have the potential to boost their learning outcomes (Narciss, 2008). In line with this, Panadero et al. (2017) showed positive effects of self-assessment interventions, where students had to monitor and self-evaluate their own work, on self-regulated learning and self-efficacy. Panadero and colleagues describe self-assessment as a core element of self-regulated learning since it supports the generation of internal feedback (Panadero et al., 2016a, 2018, for an overview). Learning diaries, for instance, can serve as instructional tool to facilitate self-assessment and to encourage reflection (Klug et al., 2011; Panadero et al., 2016b).

Fourth, feedback about the self as a person, e.g., “You are a great student,” even shows detrimental effects on students’ performance (Kluger and DeNisi, 1996) by drawing students’ attention on the self and away from the actual task. Praise at the self level has the potential to boost SRL and performance but only if students change their beliefs about the role of effort for successful learning (Hattie and Timperley, 2007). For instance, praise regarding effort or engagement (e.g., “You are a great student because you really worked hard”) can lead to increased self-efficacy for performing well (Schunk, 2012). However, praising effort alone might not suffice to foster SRL, but students need to know how to apply strategies in order to *feed forward*.

#### 1.1.4. Combining learning diaries with feedback to enhance self-regulated learning

Feedback (on all four levels) influences the process of SRL and constitutes a catalyst for change in motivation and behavior. Thus, we assume that combining internal self-regulation feedback and external process feedback should provide an ideal starting point to promote SRL. In the present study, students completed learning diaries that encouraged them to set goals and to reflect on their goal achievement after learning. That is, completing these learning diaries should guide students’ generation of internal self-regulation feedback and stimulate reflection (*feed back*) and goal setting (*feed up*). However, learning diaries alone might not be sufficient to increase SRL, since they hardly provide specific guidance on how to change SRL strategies to improve learning (*feed forward*). Hence, additional, external process feedback can facilitate monitoring and interpreting internal feedback by supporting a realistic and correct comparison between desired goals or standards (*feed up*) and the actual outcome (*feed back*). Further, explicit strategy suggestions (*feed forward*) included in the process feedback can help students to successfully adapt their strategies.

There is meta-analytic evidence that supports the assumption that feedback boosts the effects of learning diaries on achievement and motivation (Dignath et al., 2017): While studies that tested the effectiveness of learning diaries reached only a moderate average effect (Cohen’s *d* = 0.28), studies that included teacher feedback on learning diary entries yielded high effects (*d* = 0.83). However, most of these studies investigated the effect of learning diaries with open-ended items (as opposed to close-ended items), and feedback focused on outcome only. Moreover, none of these studies had used a randomized control trial. Wäschle et al. (2014) also demonstrated the power of individualized feedback on SRL, showing that externally provided visual feedback helped students to improve their time management. However, this study focused on only one aspect of SRL (time management) while neglecting other important aspects, e.g., goal setting, self-efficacy, and motivation. Hence, despite theoretical groundwork on the synergetic effects of SRL and feedback (Butler and Winne, 1995; Nicol and Macfarlane-Dick, 2006), empirical studies on the direct effects of feedback on daily reported SRL are scarce.

### 1.2. Research aims and hypotheses

Although feedback loops are an integral part in the SRL process, there is not much empirical research on how internal and external feedback interact within the SRL cycle. We want to fill this gap by investigating the effects of automatically generated individual feedback on metacognitive and motivational aspects of SRL in an experimental field study using daily morning and evening learning diaries. The ambulatory assessment via electronic learning diaries serves as part of the intervention and at the same time as fine-grained, ecologically valid measures of the SRL process (Roth et al., 2016). However, we assume that learning diaries alone as an intervention (LD) will not be sufficient to significantly improve SRL strategies over the course of five weeks. In contrast, additional process feedback (LDF) should help students to correctly monitor and adapt their learning strategies. Our design thus allows us to test whether feedback can boost positive effects of learning diaries on SRL. We chose the process level because feedback on other levels would not be feasible: Task feedback requires more context information than what is possible to collect within a learning diary, feedback about self-regulation by definition is an internal process, and feedback about the self can even be detrimental for performance.

Hence, we hypothesize that students who keep a learning diary and receive feedback on their entries will show better SRL strategies over time compared to students who keep a learning diary without receiving feedback (LD).

More specifically, we expect that group LDF will report more goal setting (**H1**), planning (**H2**), a higher self-efficacy (**H3**) and higher intrinsic motivation (**H4**) in the morning diary compared to group LD over the course of the study. Further, we hypothesize that group LDF will report a higher satisfaction with the study day (**H5**), more adherence to self-set time schedule (**H6**), higher effort (**H7**) and less procrastination (**H8**) in the evening diary compared to group LD over the course of the study. In addition to the process measures of SRL, we apply retrospective self-report questionnaires before (t1) and after (t2) the intervention period in order to test, whether feedback has an effect on trait SRL.

Second, we investigate how feedback affects daily SRL processes depending on the type of feedback. According to Narciss (2008), feedback can have reinforcing, information, or guiding function. For instance, feedback can reinforce students in their study approach (*confirmative feedback*), it can encompass information on learning outcomes or processes (*informative feedback*) or include *feed forward* on how to improve learning processes (*transformative feedback*). Therefore, we explore the effects of receiving confirmative, informative, or transformative feedback regarding self-reported planning, motivation, satisfaction with the study day, adherence to time schedule, procrastination, and effort on next days’ SRL.

Research questions, hypotheses, and methods have been preregistered via the Open Science Framework (OSF^2^) prior to conducting the study.

## 2. Method

### 2.1. Participants

Initially, *N* = 256 university students from a large university in South-Western Germany had registered for the study and were randomly assigned to either the learning diary with feedback (LDF, *n* = 129) or the learning diary without feedback (LD, *n* = 127) condition. We included only subjects into the analysis who had responded to the pre- (t1) and post- (t2) intervention questionnaires and filled in at least half of the learning diaries (18 out of 36 diary entries). Hence, the final sample consisted of *N* = 194 (LDF: *n* = 98, LD: *n* = 96; *n* = 117 female) students who were on average 22 years old (*M* = 22.21, SD = 2.72, [17; 35]). Subjects came from various fields of study, e.g., economics and political science (34%), teacher training (20%), natural sciences (20%), arts and humanities (12%), social sciences (8%), and languages (6%). On average, students were in their fourth semester (*M* = 3.91, SD = 2.59, [1, 13]). Students completed on average 30 out of 36 diary entries (*M* = 30.09, SD = 3.60, [19; 36]). As expected, the randomly assigned groups (LDF and LD) were comparable with respect to gender, age, semester, and self-reported use of SRL strategies at t1 (all *p*-values >0.05, see Table 1). Groups did not differ in their overall number of learning diaries completed. Furthermore, the number of complete diary entries was not systematically related to SRL at t1 (Pearson’s *r* between −0.11 and 0.07, all *p*-values >0.05).

**Table 1:** Baseline comparisons between experimental groups (LD, LDF) and dropout analysis.

	LDF (*n* = 98)	LD (*n* = 96)	*p*	Participants (*n* = 194)	Dropouts (*n* = 62)	*p*
Gender (*n* female)	58	59	0.365	117	30	0.158
	*M* (SD)	*M* (SD)		*M* (SD)	*M* (SD)	
Age	21.99 (2.51)	22.44 (2.93)	0.254	22.21 (2.72)	22.57 (3.14)	0.384
Semester	3.98 (2.42)	3.83 (2.77)	0.696	3.91 (2.59)	3.72 (2.27)	0.616
Planning_t1_	2.90 (1.24)	2.79 (1.27)	0.557	2.84 (1.25)	2.77 (1.09)	0.677
Self-Motivation_t1_	3.83 (1.11)	4.05 (1.20)	0.190	3.94 (1.16)	4.03 (1.25)	0.608
Self-efficacy_t1_	3.73 (0.89)	3.77 (0.95)	0.749	4.01 (0.92)	3.75 (0.76)	0.066
Reflection_t1_	3.17 (1.08)	3.32 (0.93)	0.320	3.24 (1.01)	3.17 (1.10)	0.612
Procrastination_t1_	3.71 (1.36)	3.73 (1.34)	0.945	3.72 (1.35)	3.95 (1.15)	0.233
Volition_t1_	3.03 (1.03)	3.16 (1.05)	0.353	3.09 (1.04)	2.98 (1.00)	0.465
Number diary entries	29.99 (3.52)	30.19 (3.70)	0.703	**30.09** (3.60)	**6.21** (6.62)	**<0.001**

Bolded means indicate significant group difference (two-tailed test, *p* < 0.05).

#### 2.1.1. Dropout analysis

Dropout rates were comparable in group LDF and LD (Chi^2^(1) = 0.28, *p* = 0.60). Dropouts (*n* = 62) did not differ from participants who completed the study with regard to gender, age, semester, as well as SRL strategies at t1 (all *p*-values >0.05, see Table 1). However, there was a small, yet non-significant trend that those who dropped out were more likely to report higher self-efficacy at t1 [*t*(254) = −1.85, *p* = 0.07]. Further, dropouts completed significantly fewer learning diaries compared to participants.

### 2.2. Design and procedure

**Figure 2:**
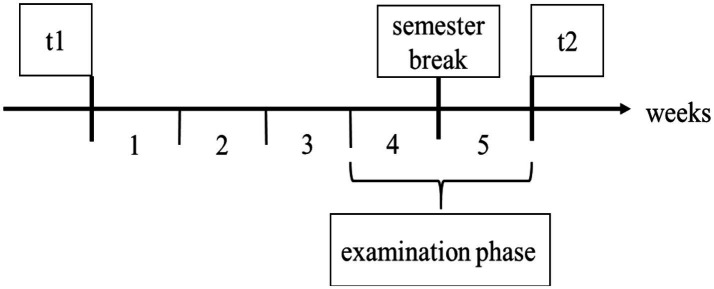
Overview survey period.

Students registered for the study online via a link to the pre-questionnaire (t1) provided via SoSci Survey^3^ (Leiner, 2019). Before starting the questionnaire, students received additional information on the study procedure and data privacy. They were informed that data will be processed anonymously, and that data will only be used for scientific purposes. After reading the terms and conditions, students gave us their informed consent and were passed on to the pre-questionnaire. When registering for the study, students were randomly assigned to one of the experimental conditions (LDF or LD). During the survey period (running 5 weeks from 15^th^ of January until 19^th^ of February, see Figure 2), students in both groups filled in daily electronic learning diaries, which comprised a morning and an evening questionnaire. Additionally, students in group LDF received daily, automated feedback throughout the whole survey period. This corresponds to a between-subjects designs (feedback vs. no feedback) with daily assessment of the dependent variables (Lischetzke et al., 2015). The day after the last learning diary has been sent out, students were asked to answer the post-questionnaire (t2) within 1 week. Students who filled in t1, t2 and completed at least 27 learning diaries (75%) received 50 € for participation.

### 2.3. Daily learning diary

The electronic learning diary as well as the feedback were implemented via SoSci Survey. Students could fill in the learning diary using their personal computer, laptop, tablet, or smartphone. Daily diaries comprised a morning questionnaire (available from 6 a.m. to 3 p.m.) and an evening questionnaire (available from 4 p.m. to 2 a.m. on the next day). Students received daily invitations via e-mail to fill in the morning and evening questionnaire, respectively. They were asked to fill in both parts every day even if they did not perform study-related tasks on a given day. The learning diary included open questions as well as closed Likert-type questions ranging on a scale from 1 (“not true”) to 6 (“true”). Making an entry in the morning and evening required about 10 minutes altogether.

The learning diary covered the forethought, performance, and self-reflection phase of the SRL circle (see Table 2; grey feedback blocks were only presented in group LDF). SRL items relevant in the forethought phase (goal setting, planning, motivation, and self-efficacy) were assessed in the morning. In the morning questionnaire, students wrote down their study goals in an open text field and indicated whether they consider these goals ambitious. Further, students reported their time goals and plans (number of hours planned for lecture and independent study time). In a last step, students indicated their study motivation and self-efficacy beliefs for that moment. Items relevant in the performance and self-reflection phase (adherence to time schedule, satisfaction with study day, procrastination, and effort) were assessed in the evening questionnaire. Lastly, students indicated whether they performed study-related tasks on that day. The last-mentioned item was used as a filter item in subsequent data analysis in order to sort students in groups that did versus did not study on that day.

**Table 2:** Overview on the daily diary items and feedback in the morning and evening questionnaire.

Morning questionnaire	
Variable	Item
Study goals	Today, I am setting myself the following study goals: [open text field]
Number of hours planned for attending lectures or courses	Today, I am planning to invest the following time for studying in-class: [open text field]
Number of hours planned for self-study	Today, I am planning to invest the following time for self-study: [open text field]
Goal setting	Today, I am setting myself ambitious goals. [Likert-type]
Planning	Today, I have a specific plan, according to which I will perform today’s study-related tasks. [Likert-type]
LDF: Students received adaptive *feed forward*, if they reported low planning on the previous item (“1” or “2” on 6-point scale): “Please check your timetable: How much time do you need to complete the tasks on your to-do-list? Have you added a time-buffer for unexpected events? Please sort your tasks according to their importance and urgency.”	
Intrinsic Motivation	Today, I study because I enjoy the topics. [Likert-type]
LDF: Students received adaptive *feed forward*, if they reported low motivation on the previous item (“1” or “2” on 6-point scale): “Please reflect why today’s learning topics are relevant and useful for you.”	
Self-efficacy	Today, I know how to proceed to have a successful study day. [Likert-type]
Evening questionnaire	
LDF: Students were shown their goals and time plans they made in the morning (*feed up*): In the morning, you set the following study goals: [open text field of morning diary was displayed] In the morning, you planned to invest: [number of hours] for self-study and [number of hours] for studying in-class.	
Satisfaction with study day	I am satisfied with what I have achieved today (study-relevant). [Likert-type]
Adherence to time schedule	Today, I adhered to my time schedule. [Likert-type] + Outside Option: “I have not made myself a time schedule today.”
Procrastination	Today, I have postponed unpleasant tasks. [Likert-type]
Effort	Today, I invested effort while studying (in-class and self-study) [Likert-type]
LDF: Students received written feedback on satisfaction with study day, adherence to time schedule, procrastination and effort (*feed back* and *feed forward* formulation see Table 3)	
Study day	Did you perform study-related tasks today? (1 = “yes”; 0 = “no”)

LDF, Learning diary with feedback. Grey blocks were only presented in group LDF.

### 2.4. Experimental manipulation: feedback

Students in the feedback condition received additional, daily feedback which provided information on goals (*feed up*), current performance (*feed back*), and suggestions how to close the gap between current performance and goals (*feed forward*). The feedback intervention consisted of three components. First, in the morning questionnaire, students received adaptive *feed forward* if they reported low intrinsic motivation or planning (see Table 2). Second, in the evening questionnaire, students were shown their study goals and time goals they had set themselves in the morning (*feed up*) in order to facilitate their generation of internal feedback in terms of satisfaction ratings. Third, students indicated their satisfaction with the study day, adherence to self-set time schedule, procrastination, and effort. In case of the adherence to self-set time schedule, students had the additional option to indicate that they did not make a time schedule, which corresponds to the “no schedule” feedback.

Based on their self-reports, written feedback on their satisfaction with the study day, adherence to self-set time schedule, effort and procrastination was provided (*feed back*). If someone indicated high values on the respective scales (“5” or “6” on a 6-point Likert scale), *confirmative* feedback was provided which reinforced students’ regarding their study approach. Students who reported medium (“3″ or “4″) values on the respective scales received *informative* feedback indicating that there is room for improvement while students reporting low (“1″ or “2″) values received *transformative* feedback which encompassed an additional strategy suggestion (*feed forward*). Table 3 provides an overview of all possible feedback sentences. Feedback was generated automatically by Sosci Survey based on students’ self-reports and was provided daily throughout the whole survey period (36 days).

**Table 3:** Overview on daily written feedback provided in the evening questionnaire on satisfaction with study day, adherence to time schedule, procrastination, and effort.

Variable	Feedback	
Satisfaction with study day	Confirmative feedback [“5” or “6” on 6-point scale]	You are very satisfied with what you have achieved today.
	Informative feedback [“3” or “4” on 6-point scale]	You are somewhat satisfied with what you have achieved today.
	Transformative feedback [“1” or “2” on 6-point scale]	You are not satisfied with what you have achieved today. Try to set yourself goals for tomorrow. That’s motivating!
Adherence to time schedule	Confirmative feedback [“5” or “6” on 6-point scale]	You perfectly adhered to your time schedule. You know how much time you need to achieve your goals. Very good!
	Informative feedback [“3” or “4” on 6-point scale]	You only partially adhered to your time schedule today. When did you deviate from your timetable and why?
	Transformative feedback [“1” or “2” on 6-point scale]	You did not adhere to your time schedule today. Which tasks took more or less time than expected?
	Transformative feedback [“0” Outside option: I have not made myself a time schedule today.]	You did not make yourself a time schedule today. Try to make yourself a To-Do list tomorrow and think about how much time you need to achieve each goal on your list.
Procrastination	Confirmative feedback [“5” or “6” on 6-point scale]	You did not postpone unpleasant tasks today. Very good!
	Informative feedback [“3” or “4” on 6-point scale]	You postponed some unpleasant tasks today. Maybe, you can try to reward yourself if you have reached your goals?
	Negative Feedback [“1” or “2” on 6-point scale]	You postponed unpleasant tasks today. Try to divide your goals into smaller sub goals tomorrow.
Effort	Confirmative feedback [“5” or “6” on 6-point scale]	You invested a lot of effort while working today. Very good!
	Informative feedback [“3” or “4” on 6-point scale]	You invested some effort while working today, but there is room for improvement!
	Transformative feedback [“1” or “2” on 6-point scale]	You did not invest much effort while working today. You can do more!

For feed forward suggestions, we used strategies that were part of empirically tested SRL trainings (Schmitz and Wiese, 2006; Bellhäuser et al., 2016; Dörrenbächer and Perels, 2016). For example, to foster intrinsic motivation we applied the utility-value-intervention approach based on Wigfield and Eccles (2000). In this approach, participants are instructed to write down reasons why a certain learning topic is relevant and useful for attaining their personal goals which should increase intrinsic motivation for studying this topic (van der Beek et al., 2020).

### 2.5. Measures

All of the self-report measures presented below were assessed on a six-point Likert scale ranging from “not true” to “true.”

#### 2.5.1. Daily self-regulated learning: state measures

The morning questionnaire contained SRL items on goal setting, planning, motivation, and self-efficacy. Satisfaction with the study day, adherence to time schedule, procrastination, and effort were assessed in the evening questionnaire (see Table 1). We adopted SRL items from a previous diary study by Liborius et al. (2019). We used single items to assess each of the abovementioned variables, which is common in studies that use daily assessment in order to assure participants commitment with the repeated measurements (Bolger et al., 2003; Fisher and To, 2012). The diary variables (aggregated across all measurement points) correlated substantially with the corresponding trait variables at t1 (e.g., state procrastination with trait procrastination: *r* = 0.43, *p* < 0.001), indicating validity of the state measures (see Table 4).

**Table 4:** Reliabilities and correlations among SRL variables (t1) and diary variables measured in the morning or evening questionnaire.

	Variables	*M* (SD)	ω (t1)	ω (t2)	1	2	3	4	5	6	7	8	9	10	11	12	13	14
	Person level																	
1	Study satisfaction_t1_	4.43 (0.44)	0.83	−	−													
2	Planning_t1_	2.85 (1.25)	0.80	0.83	0.05													
3	Self-Motivation_t1_	3.94 (1.16)	0.68	0.69	0.06	0.30***												
4	Self-efficacy_t1_	3.75 (0.92)	0.90	0.90	0.43***	0.10	0.32***											
5	Reflection_t1_	3.24 (1.01)	0.71	0.73	0.11	0.39***	0.39***	0.23**										
6	Procrastination_t1_	3.72 (1.35)	0.94	0.94	−0.23**	−0.35***	−0.30***	−0.46***	−0.24***									
7	Volition_t1_	3.09 (1.04)	0.82	0.80	0.22**	0.26***	0.44***	0.51***	0.23**	−0.46***								
	Daily level																	
8	Goal setting_m_	4.76 (0.61)			0.06	0.16*	0.08	0.09	0.08	−0.08	0.06							
9	Planning_m_	4.20 (0.99)			0.35***	0.31***	0.19**	0.13	0.20**	−0.30***	0.35***	0.31***						
10	Intrinsic Motivation_m_	3.81 (0.90)			0.43***	0.16*	0.16*	0.30***	0.13	−0.20**	0.43***	0.20**	0.47***					
11	Self-efficacy_m_	4.22 (0.79)			0.38***	0.25***	0.13	0.30***	0.20**	−0.27***	0.38***	0.27***	0.64***	0.56***				
12	Satisfaction with study day_e_	3.87 (0.73)			0.36***	0.23**	0.10	0.35***	0.07	−0.37***	0.36***	0.19**	0.41***	0.53***	0.54***			
13	Adherence to time schedule_e_	3.32 (1.32)			0.31***	0.26***	0.18*	0.11	0.17*	−0.30***	0.31***	0.24***	0.73***	0.38***	0.39***	0.58***		
14	Procrastination_e_	2.99 (0.87)			−0.27***	−0.20**	−0.01	−0.24***	−0.01	0.43***	−0.27***	−0.10	−0.26***	−0.30***	−0.32***	−0.58***	−0.36***	
15	Effort_e_	4.38 (0.70)			0.26***	0.22**	0.19**	0.23**	0.10	−0.34***	0.27***	0.46***	0.42***	0.39***	0.51***	0.67***	0.44***	−0.52***

*p < 0.05, **p < 0.01, ***p < 0.001 (two-tailed). _t1_ Scale assessed in the pre-questionnaire at t1. _m_ Item assessed in the morning questionnaire. _e_ Item assessed in the evening questionnaire.

#### 2.5.2. Self-regulated learning: trait measures

We measured trait SRL strategies before (t1) and after the intervention period (t2) by means of self-report questionnaires. We used the short SRL questionnaire for university students (SRL@U, Bellhäuser and Schmitz, 2017) to assess goal setting (4 items, i.e., “I set myself challenging goals.”), self-motivation (3 items, i.e., “I think of past success to increase my motivation.”), reflection (4 items, i.e., “At the end of the day, I ask myself whether I am satisfied with my performance.”), and volition (4 items, i.e., “I can bring myself in the right mood for studying.”). Planning (3 items, i.e., “While studying, I adhere to a specific time plan.”) was measured using the German Learning Strategies Inventory (LIST; Wild and Schiefele, 1994), which is the German version of the Motivated Strategies for Learning Questionnaire (MSLQ; Pintrich et al., 1991). We assessed self-efficacy using an adapted version of the Professional Self-efficacy Scale (Schyns and von Collani, 2014) that consists of nine items by rephrasing the items such that they refer to university education (e.g., “When I am confronted with a problem in my studies, I can usually find several solutions.”). We computed an overall self-efficacy score by taking the average of the nine positively coded items. Procrastination was measured using the Procrastination Questionnaire for Students (PFS, Glöckner-Rist et al., 2009). Values on the seven items were averaged to one overall score whereby larger values indicate a higher degree of procrastination. On average, omega (McDonald, 1999) indicated satisfying internal consistencies of the subscales (*ω* = 0.68 to *ω* = 0.94, see Table 4) except for goal setting (t1: *ω* = 0.65, t2: *ω* = 0.57). Therefore, the subscale on goal setting (trait measure, not state measure) was excluded from further analyses.

#### 2.5.3. Study satisfaction

Students’ overall satisfaction with their course of studies was assessed at t1 via a five item scale (adopted from Liborius et al., 2019), e.g., “I am very satisfied with my course of studies” (*ω* = 0.83).

#### 2.5.4. Grades

At t2, students reported their grades in their written and oral exams to the best of their knowledge up to that point. The average GPA was 2.3 (SD = 0.90), whereby lower grades represent higher performance in the German grading system. Type and number of exams varied to a large degree since students came from very heterogeneous fields of study. Therefore, grades were hardly comparable, since demands and grading of the various exams differed to a great extent. Further, at t2, many students have not yet received their grades which caused a substantial number of missing entries. We obtained at least one grade from 132 out of the 194 students (69%), but due to a lack of comparability of grades across study fields, we refrained from using grades as outcome variable.

### 2.6. Missing data and data exclusion criteria

The final sample consisted of 194 participants who answered the questionnaires at t1 and t2 as well as filled in at least half of the in daily learning diaries over a period of 36 days. Hence, the maximum number of observations that could be obtained for each of the diary variables was 194 subjects*36 days = 6,984. Missing data ranged between 7 and 11% (*M* = 9.23, SD = 0.02) for the eight diary variables. In multilevel analysis, observed data on level 1 (daily level) are used to define a vector for each person (level 2) based on maximum likelihood estimates of the means and variance–covariance matrices (Raudenbush and Bryk, 2002). Since missing entries are not considered for estimation, we did not impute the missing data for the analyses presented below. Moreover, we excluded diary entries, if students did not set themselves goals in the morning and further indicated that they did not perform any study-related tasks in the evening, i.e., they took a day off. On these days, student neither planned to study nor actually studied but had to respond to the learning diary. Hence, their ratings on SRL strategies and goal achievement do not contain meaningful information. For instance, students reported less ambitious study-related planning on non-study days which, however, does not imply worse self-regulation. According to this criterion, 890 out of 6,984 possible entries (13%) were excluded from further analysis.

### 2.7. Multilevel analysis

To investigate the effects of feedback on daily self-regulated learning, we conducted multilevel analysis, whereby time points (days, level 1) are clustered within subjects (level 2). Multilevel modelling, also known as hierarchical linear modelling (Raudenbush and Bryk, 2002), takes into account that observations that originate from one person cannot be assumed to be independent of each other. Next to such statistical independency, longitudinal multilevel modelling accounts for the temporal dependency. Observations that are closer to each other with respect to the temporal order of measurement are assumed to be more similar than observations far apart in time, which can bias level 1 variances (Bliese and Ployhart, 2002). Hence, we specified a first-order autoregressive error structure to take into account any possible autocorrelation. We used Stata 15.1 (StataCorp, 2017) for data analysis.

A step-wise procedure was used to specify each multilevel model (Bliese and Ployhart, 2002; Raudenbush and Bryk, 2002). First, the unconditional means model was computed that included only the dependent variables. Second, we analyzed whether the experimental manipulation (feedback) had an effect on daily SRL. Following recommendations for the evaluation of daily interventions (Lischetzke et al., 2015), we specified multilevel models using group (1 = LDF, 0 = LD) as a between person predictor of daily SRL while controlling for baseline levels of the respective trait variable at t1. Further, random time slopes (*σ*^2^) account for within subject variability over time. A significant, positive effect of the feedback variable would indicate a main effect, i.e., a higher overall value of the respective dependent variable in group LDF compared to group LD. Hence, we examined mean-level changes in the dependent variables to evaluate the general effectiveness of the feedback intervention (Lischetzke et al., 2015).

## 3. Results

### 3.1. Effects of individual feedback on daily self-regulated learning

According to our research goal, we investigated whether feedback affects daily SRL. We hypothesized that feedback will have a positive effect on the overall use of SRL strategies over the course of the study.

Table 5 shows the average persons’ mean level across the observation period and provides estimates of the variability on level 1 (within subjects) and level 2 (between subjects). The interclass coefficient (ICC) represents the percentage of variance that lies between subjects, indicating that approximately 17 to 47% of the variance in the dependent variables was between subjects, while 53 to 83% of variance was within subjects. The within subject variability over time was higher than the between subject variance for all variables.

**Table 5:** Unconditional means models for dependent variables.

	Mean	Variance between (*τ*)	Variance within (*σ*^2^)	ICC	Number of observations
Dependent variables (morning)					
Goal setting	4.73	0.61	0.83	0.42	5,868
Planning	4.19	0.91	1.07	0.46	5,868
Self-efficacy	4.55	0.60	0.67	0.47	5,868
Intrinsic motivation	3.77	0.71	0.96	0.42	5,868
Dependent variables (evening)					
Satisfaction	3.81	0.34	1.64	0.17	5,562
Adherence to time schedule	3.34	1.52	2.82	0.35	5,562
Procrastination	3.01	0.70	2.18	0.24	5,562
Effort	4.37	0.53	1.50	0.26	5,562

*N* = 194. The mean represents an average person’s mean value across the 36 points of measurement. The ICC represents the proportion of variance between persons.

**Figure 3:**
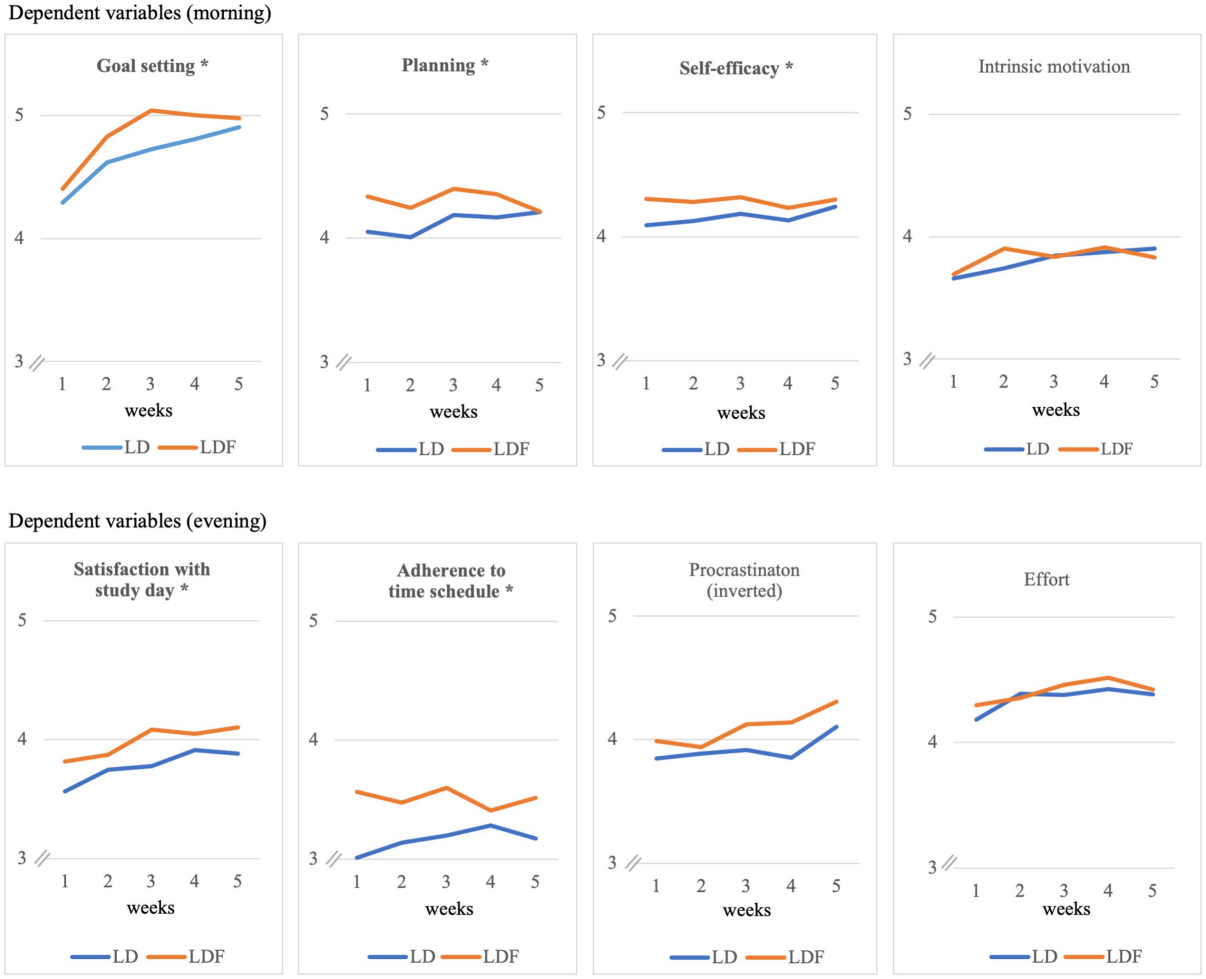
Development of SRL variables over the course of the study in group with (LDF) and without (LD) additional feedback. Feedback was provided throughout the whole survey period starting at day 1. Bold headings indicate that average group difference (LD vs. LDF) is significant (*p < 0.05).

Table 6 provides an overview of the results of the multilevel analyses. Figure 3 graphically shows the average development of the dependent variables over time separately for group LD and group LDF. Note that feedback has been provided throughout the whole survey period, already beginning on day 1. As can been seen in the plots, the experimental group LDF showed higher values in every dependent variable over almost the entire period of the study.

**Table 6:** Random intercept models with feedback condition (0 = LD, 1 = LDF) and outcome at baseline (t1) as predictors of daily SRL.

Dependent variables (morning)												
	Goal setting			Planning			Self-efficacy			Intrinsic motivation		
	Estimate	SE	*R* ^2^	Estimate	SE	*R* ^2^	Estimate	SE	*R* ^2^	Estimate	SE	*R* ^2^
Fixed effects												
Level 2 (Between)			0.08			0.19			0.08			0.07
Intercept	3.98***	0.20		3.36***	0.17		3.51***	0.23		3.14***	0.23	
Feedback	0.19*	0.08		0.24*	0.11		0.24*	0.11		0.09	0.12	
Outcome at baseline (*t*1)	0.15**	0.05		0.26***	0.05		0.24***	0.06		0.15**	0.05	
Random parameters												
Residual variance (*τ*)	0.53	0.07		0.72	0.09		0.52	0.06		0.64	0.08	
Level 1 (Within)												
Residual variance (*σ*^2^)	0.85	0.02		1.05	0.02		0.68	0.01		0.98	0.02	
Autocorrelation	0.16	0.02		0.16	0.02		0.13	0.02		0.12	0.02	

Dependent variables (evening)												
	Satisfaction with study day			Adherence to time schedule			Procrastination			Effort		
	Estimate	SE	*R* ^2^	Estimate	SE	*R* ^2^	Estimate	SE	*R* ^2^	Estimate	SE	*R* ^2^
Fixed effects												
Level 2 (Between)			0.09			0.32			0.22			0.07
Intercept	2.65***	0.19		2.28***	0.23		2.05***	0.17		3.76***	0.16	
Feedback	0.20*	0.09		0.40*	0.17		−0.17	0.11		0.18	0.05	
Outcome at baseline (t1)	0.24***	0.04		0.30***	0.07		0.28***	0.04		0.09***	0.09	
Random parameters												
Residual variance (τ)	0.25	0.05		1.20	0.17		0.48	0.09		0.46	0.07	
Level 1 (Within)												
Residual variance (σ^2^)	1.66	0.03		2.86	0.06		2.21	0.05		1.52	0.03	
Autocorrelation	0.07	0.02		0.13	0.02		0.12	0.02		0.10	0.02	

*p < 0.05, **p < 0.01, ***p < 0.001.

In line with our hypothesis, we found significant main effects of feedback in most of the dependent variables. The process data in group LDF showed on average more ambitious goal setting (*b* = 0.19, **H1**), better planning (*b* = 0.24, **H2**), self-efficacy (*b* = 0.24, **H3**), satisfaction with the study day (*b* = 0.20, **H5**), and adherence to self-set time schedule (*b* = 0.40, **H6**) (all *p*-values below 0.05; see Table 6) than group LD. Intrinsic motivation (**H4**), effort (**H7**), and procrastination (**H8**) were not affected by feedback. Following the convention by Funder and Ozer (2019), effect sizes of *b* = 0.10 can be labeled as *small*, *b* = 0.20 as *medium*, and *b* = 0.30 as *large.*

### 3.2. Explorative analyses of effects of confirmative, informative and transformative feedback on next day’s self-regulated learning

The analysis above investigated the effects of the feedback intervention on average self-reported SRL strategies across the whole survey period. However, in order to provide further insights on how feedback on a given day affects SRL on the subsequent day, we conducted exploratory follow-up analyses.

We tested whether presenting confirmative, informative, or transformative feedback regarding planning and motivation (reported in the morning) as well as satisfaction with the study day, adherence to time schedule, procrastination, and effort (reported in the evening) affected next day’s response in this particular SRL variable. For this purpose, we created dummy variables indicating which type of feedback (confirmative, informative, or transformative) has been shown, and built an interaction term between type of feedback and experimental condition (LD vs. LDF). For instance, to test the effect of presenting positive feedback regarding effort on day *t* on next day’s effort (*t* + 1), we only analyzed those observations in group LDF and LD, in which students responded that they invested high effort (“5” or “6” on six-point scale). Hence, we compared subjects in group LDF who received confirmative feedback regarding effort with those observations in group LD who would have received confirmative feedback if they would have been assigned to the feedback condition. This allowed us to compute the main effect of receiving confirmative feedback regarding effort on next day’s effort while controlling for previous effort. Note that the design no longer constitutes a purely randomized controlled trial since the assignment to a criterion group depends not only on the experimental design but also on the occurrence of certain responses in the learning diary. Results of the multilevel analyses including the number of valid observations in group LD and group LDF used for the respective analysis are presented in Table 7.

**Table 7:** Main effects of receiving confirmative, informative or transformative feedback on next day’s goal setting, planning, satisfaction with study day, adherence to time schedule, procrastination and effort.

	Number of observations				
	LD	LDF	Coefficient	SE	*p*
Dependent variable (morning): Planning _t + 1_					
Transformative feedback _t_	1,668	1,736	0.26**	0.09	0.003
Dependent variable (morning): Motivation _t + 1_					
Transformative feedback _t_	1,473	1,444	0.10	0.07	0.152
Dependent variable (evening): Satisfaction with study day _t + 1_					
Confirmative feedback _t_	964	1,190	0.30**	0.09	0.001
Informative feedback _t_	1,288	1,152	0.05	0.09	0.590
Transformative feedback _t_	396	223	1.53**	0.49	0.002
Dependent variable (evening): Adherence to time schedule _t + 1_					
Confirmative feedback _t_	1,041	1,223	0.32*	0.13	0.016
Informative feedback _t_	865	845	0.12	0.09	0.191
Transformative feedback _t_	552	572	0.20*	0.10	0.045
Transformative feedback _t_ (no time schedule)	507	412	0.59***	0.11	< 0.001
Dependent variable (evening): Procrastination _t + 1_					
Confirmative feedback _t_	1,237	1,348	−0.23**	0.06	0.002
Informative feedback _t_	915	897	−0.01	0.07	0.981
Transformative feedback _t_	524	507	−0.04	0.22	0.883
Dependent variable (evening): Effort _t + 1_					
Confirmative feedback _t_	1,463	1,470	0.08	0.06	0.157
Informative feedback _t_	972	1,055	0.04	0.07	0.521
Transformative feedback _t_	151	314	−0.11	0.09	0.216

Confirmative feedback was given if students indicated “5” or “6” on six-point Likert scale. Informative feedback was given if students indicated “3” or “4” on six-point Likert scale. Transformative feedback was given if students indicated “1” or “2” on six-point Likert scale. In case of adherence to time schedule, students also received transformative feedback if they reported that they did not make a time schedule. In the morning, transformative feedback was provided adaptively if students indicated low planning or low motivation (values below “5” on six-point Likert scale).

*p < 0.05, **p < 0.01 ***p < 0.001.

Students who received transformative feedback regarding their planning in the morning reported having more concrete plans on the subsequent day compared to students in group LD who did not receive feedback. Transformative motivational feedback did not predict higher motivation on the next day. Regarding feedback on satisfaction with the study day, students reported higher satisfaction after receiving either confirmative or transformative feedback compared to no feedback. Informative feedback did not affect next day’s satisfaction. With respect to feedback on the adherence to the self-set time schedule, confirmative feedback, transformative feedback, and feedback after no time schedule has been made showed significant positive effects on next day’s adherence to time schedule compared to no feedback. Informative feedback did not significantly affect next days’ adherence to time schedule. Regarding procrastination, only confirmative feedback had significantly predicted next day’s procrastination, which means that procrastination further decreased after confirmative feedback compared to no feedback. None of the other feedback types significantly affected next day’s procrastination. As already visible in Table 6, students did not report changes in self-reported effort due to the provision of feedback.

### 3.3. Effects of individual feedback on trait self-regulated learning

To test whether feedback has an effect on the SRL trait measures, a repeated multivariate analysis of variance (MANOVA) with time as a repeated within-subject measure (t1, t2) and feedback as between-subject factor (1 = LDF, 0 = LD) was conducted. As dependent variables, we included all SRL subscales, except for goal setting which was excluded due to insufficient internal consistency. The results yielded a significant main effect of time [*F*(7, 186) = 10.97, *p* < 0.001, *η*^2^ = 0.29]. Following the convention by Funder and Ozer (2019) this can be referred to as a very large effect. Irrespective of the feedback condition, all students reported more planning, and reflection activity, and more self-motivation, volition, and higher self-efficacy, and reduced procrastination at t2 compared to t1 (see Table 8). However, contrary to our hypothesis, no interaction effect of feedback condition and time was reflected in the SRL questionnaire data [*F*(7, 186) = 1.04, *p* = 0.41, *η*^2^ = 0.04]. Both groups (LD and LDF) reported a similar change in SRL, self-efficacy and procrastination over time.

**Table 8:** Multivariate analysis of variances (MANOVA).

		*t*1	*t*2	Time	*η* ^2^	Feedback		Feedback × Time	
		*M* (SD)	*M* (SD)	*F* (1, 192)		*F* (1, 192)	η^2^	*F* (1, 192)	η^2^
Planning	LD	2.79 (1.27)	3.05 (1.33)	17.53**	0.08	1.58	0.01	1.28	0.01
	LDF	2.90 (1.24)	3.35 (1.23)						
Self-motivation	LD	4.05 (1.20)	4.58 (0.99)	20.99**	0.20	2.34	0.01	0.01	< 0.01
	LDF	3.83 (1.11)	4.38 (1.12)						
Self-efficacy	LD	3.77 (0.95)	4.02 (0.91)	14.49**	0.07	0.64	< 0.01	1.10	0.01
	LDF	3.73 (0.89)	3.87 (0.88)						
Volition	LD	3.16 (1.05)	3.37 (1.06)	25.41**	0.12	0.11	< 0.01	2.53	< 0.01
	LDF	3.03 (1.03)	3.42 (0.95)						
Reflection	LD	3.32 (0.93)	3.42 (0.99)	5.80*	0.03	0.57	< 0.01	0.98	0.01
	LDF	3.17 (1.08)	3.43 (0.98)						
Procrastination	LD	3.73 (1.34)	3.41 (1.27)	35.66**	0.16	0.85	< 0.01	0.15	< 0.01
	LDF	3.71 (1.36)	3.35 (1.32)						

LD = Learning diary without feedback (*n* = 96). LDF = Learning diary with feedback (*n* = 98).

*p < 0.05, **p < 0.01.

## 4. Discussion

In the present study, we investigated the effects of automatically generated, adaptive feedback on SRL in an experimental field study using daily learning diaries. Almost 200 students in two experimental conditions (with or without additional feedback) reported their application of SRL strategies in the morning and in the evening over the course of 36 days, which allowed us to investigate SRL processes using a rich, longitudinal dataset. Data acquisition took place in the natural learning environment and during the critical examination phase at the end of semester, which ensures high ecological validity of the daily diary data.

According to our research aim, we investigated the effects of process feedback on SRL using multiple data sources: diary data and pre-and post-questionnaires. Analysis of the daily diary data revealed medium-sized effects of feedback on process data about goal setting, planning, self-efficacy, satisfaction with the study day, and a large effect on adherence to self-set time schedule. Moreover, exploratory analysis of specific feedback sentences showed differential effects depending on the type of feedback. In short, transformative feedback including *feed forward* and confirmative feedback predicted better SRL on the subsequent day compared to no feedback, whereas informative feedback did not predict next day’s SRL. Pre-post comparisons of students’ self-reported general use of SRL strategies did not show any effects of feedback on trait SRL.

### 4.1. Does feedback improve self-regulated learning?

According to theoretical models of SRL (Butler and Winne, 1995; Zimmerman, 2002), we hypothesized that learning diaries with individual process feedback can help students reflecting on their study behavior and improve SRL. The multimodal assessment applied in this study yielded different results regarding the effectiveness of feedback.

The results of the pre-post comparison showed that students in both groups reported significantly better general planning, self-motivation, self-efficacy, volition, and reflection from t1 to t2 and reduced their self-reported general procrastination over time. The overall time effect was large, especially for general self-motivation, procrastination, and volition (Cohen, 1992). One explanation is that the learning diary constituted an effective intervention itself, irrespective of feedback provision. This would be in line with the idea that learning diaries promote SRL by stimulating monitoring and reflection on the own study process (Schmitz and Perels, 2011), but in contrast to previous research, which did not find effects of learning diaries on SRL (Fabriz et al., 2014; Bellhäuser et al., 2016; Dörrenbächer and Perels, 2016; Bellhäuser et al., 2022). However, since we did not include a control group without learning diary, we do not know how SRL would have developed over time without the use of learning diaries. The study took place before and during the examination phase at the end of semester. Hence, the increase in SRL strategies and especially the decrease in procrastination might also be caused by the fact that students could no longer postpone their studying due to important deadlines (Theobald et al., 2018). In contrast to our hypothesis, the pre-post comparison of the trait SRL measures indicated neither a main effect of feedback on SRL, nor an interaction of feedback and time. It might be that the huge overall time effect covered the (probably rather small) effect of feedback on SRL.

By contrast, the analysis of the process data were in line with our assumption, indicating a significant positive main effects of feedback on daily-reported goal setting, planning, self-efficacy, satisfaction with the study day, and adherence to self-set time schedule. Thus, students in the feedback condition set more ambitious goals, reported to make better plans in the morning, and indicated higher self-efficacy in their own competences to achieve those plans. In the evening, careful planning paid off and students were more likely to report successful adherence to their time schedule, which might also explain an increased satisfaction with the study day (Liborius et al., 2019). This is in line with the results of Wäschle et al. (2014), who found positive effects of visual feedback on time management skills.

We did not find process feedback to affect intrinsic motivation, effort, nor procrastination. All of these SRL components belong to the motivational and volitional part of SRL. Apart from the possibility that this finding is only a random finding, one explanation might be that the feedback intervention did not sufficiently target these motivational SRL components, but rather metacognitive components since the diary focused on planning. It could also be the case that motivation and volition are more difficult to tackle by means of interventions compared to metacognitive strategies (e.g., planning). Consistent with this, Theobald and Bellhäuser (2022) also found a positive effect of a feedback intervention on metacognition but not on motivation. Dignath and Büttner (2008) also found that older students especially benefitted from SRL interventions that targeted metacognitive strategies and reflection. Besides that, self-reported effort was on average quite high over the course of the study. In this study, students usually indicated that they invested a lot of effort, which could also be an indication of ceiling effects or overconfidence (Dunlosky and Rawson, 2012).

Taken together, the results from our multimodal assessment (SRL as state vs. trait measure) provide conflicting results to the question whether process feedback improves SRL. While the SRL questionnaire that was used in the pre-and posttest gives an indication on how students estimate their general use of SRL strategies (SRL as trait), the process data from the learning diary indicates how students estimate their situative use of SRL strategies (state). Hence, we argue that individual and daily feedback has the potential to foster situative SRL and adds to the exclusive use of learning diaries.

Furthermore, these results contribute to the question on how to assess SRL in a valid way. Clearly, a questionnaire that assesses SRL strategy use generally as a trait and a learning diary that assesses SRL states in a situative way, measure SRL in two different ways. Trait-like self-report questionnaires are frequently criticized since they lack situation specificity and do not necessarily reflect student’s actual study behavior in a given situation (Winne et al., 2002; Roth et al., 2016; Panadero et al., 2016b). In contrast, daily learning diaries (although still based on self-report) offer a more context sensitive measure of the SRL process, which complies with the dynamic nature of SRL (Schmitz and Wiese, 2006). Thus, learning diaries offer high ecological validity, since learners complete the diaries in their natural learning environment (Klug et al., 2011). As the feedback has been very specific for each single day of the intervention, it seems likely that learning diaries captured the small adaptations in daily SRL better than general SRL questionnaires that assess SRL as a trait and might be less sensitive to the treatment.

One more explanation for the lack of feedback effect on the trait measures might be the design of our study: Longer interventions have been shown to have larger effects (Dignath and Büttner, 2008), so the 4 weeks of our study may have been too short. Also, changes in SRL traits might show only after a certain delay (Cousins et al., 2022) and without a follow-up measurement we might have missed this effect. Further, the effect of the feedback diary might be larger when combined with a training intervention (Bellhäuser et al., 2022).

Researchers should further investigate how state and trait measures of SRL are associated, and how we can assess SRL more precisely. As we showed in Table 4, daily state measures of SRL are correlated with their respective trait measures, but these correlations are far from perfect—leaving room for many other sources of influence (e.g., biased self-perception in the trait questionnaire). Multimodal assessment is a first step into disentangling the existing assessment methods in the field of SRL research.

### 4.2. What kind of feedback is most effective?

In the present study, we chose the level of process feedback (Hattie and Timperley, 2007) as our target because it can be produced without detailed context knowledge (unlike outcome feedback that is heavily dependent on the specific task). Contrary to internal self-regulation feedback (which by definition is created internally by the learner), it is possible to manipulate process feedback experimentally. Finally, in contrast to the self level of feedback (which can be detrimental for learning outcomes), process feedback has been shown to foster self-regulated learning (Schunk, 2012).

With respect to the specific type of feedback (confirmative, informative, transformative), our exploratory analyses showed that feedback which confirmed students in their study approach or which included *feed forward* (transformative feedback) showed larger effects than informative feedback. These results have to be interpreted cautiously due to the fact that they are not based on a purely randomized experimental design. Whether students received confirmative, informative, or transformative feedback depended foremost on what they reported in their diary about their learning behavior. However, only students in the experimental group received such feedback. Also, we compared the impact of feedback only to those days, on which students in the control group would have received the same feedback, given they had been assigned to the other experimental condition. Therefore, we believe that the following results can be interpreted as initial evidence that needs confirmation in future research:

Regarding feedback on satisfaction with the study day, confirmative and transformative feedback resulted in higher satisfaction on the subsequent day compared to no feedback. When students received transformative feedback, they simultaneously received a recommendation on how to *feed forward*. They were advised to set themselves goals for tomorrow, which revealed to be highly effective. For students, who had a good study day, generating positive internal feedback and external confirmative feedback reinforced them in their learning approach. In contrast, the system reported back to students with average satisfaction that there is room for improvement (informative feedback). This feedback did not offer enough guidance on how to *feed forward*.

The same reasoning holds for feedback on adherence to self-set time schedule and the transformative feedback regarding planning provided in the morning. Confirmative feedback reassured students that they are on the right track and might even contributed to increased self-efficacy in their own capabilities. The transformative feedback was combined with the provision of a concrete strategy on how to proceed in order to enhance time management (i.e., reflecting on which tasks took longer than expected or making a To-Do list the next day). The informative feedback only provided a non-directive reflective prompt (When did you deviate from your timetable and why?). Reflective prompts can be helpful if students are willing to engage in deeper reflective processes and already know adequate learning strategies to adapt their study behavior accordingly (Wirth, 2009). Otherwise, reflective prompts are too unspecific to guide students on how to *feed forward*.

Regarding procrastination, only confirmative feedback significantly decreased next day’s procrastination compared to no feedback. Neither informative nor transformative feedback showed an effect, although both feedback sentences encompassed a strategy on how to *feed forward*. One explanation might be that these strategy recommendations (rewarding oneself or dividing goals into smaller sub goals) did not help reducing procrastination, or students did not apply the strategies. Moreover, while reasons for poor satisfaction with self-set goals and time schedules are rather straightforward, reasons for procrastination are manifold (Steel, 2007) and can originate from low self-efficacy (Wäschle et al., 2014), poor time management (Grunschel et al., 2013), or more stable personal characteristics, e.g., low conscientiousness (Theobald et al., 2018). Therefore, providing appropriate strategy recommendations would require a more detailed assessment of the specific reasons for current procrastination. This might also explain why feedback did not affect effort. According to the missing overall effect of feedback on effort, there was no effect of specific feedback sentences on next day’s effort compared to no feedback. As already known from previous literature, effort praise is only effective when it leads to a change in students understanding of the role of effort for successful learning (Schunk, 2012). Otherwise, effort praise provides little guidance on how to *feed forward*, which makes it largely ineffective (Hattie and Timperley, 2007).

To sum up, confirmative and transformative feedback seems to support students’ monitoring and evaluation and might help them to draw conclusions for the next learning process in terms of a feedback loop (see Zimmerman, 2002). Confirmative feedback seems to encourage students that they are studying the right way and may lead to increased confidence and self-efficacy to do well. Further, confirmative feedback might guide students’ attention towards behavior that they already implemented but maybe did not notice consciously, thereby helping them to maintain this behavior. Transformative feedback has the potential to support students if it is combined with effective strategy recommendations on how to *feed forward*. Effort praise alone did not affect SRL.

### 4.3. How should we feed forward?—study limitations and ideas for future research

Although the present study offers important insights on how feedback shapes daily SRL, future studies should address some research limitations.

First, we did not include a control condition without learning diaries, since we did not intend to investigate the effectiveness of learning diaries. Unexpectedly, pre-post comparisons showed that self-reported SRL significantly increased from t1 to t2. We do not know whether SRL improved due to the diary in terms of an intervention, or whether SRL increased because of the approaching examination phase, or whether the increase is a measurement artifact due to participants’ habituation to the questionnaire items. Future studies should therefore explore how self-reported SRL develops over the course of a semester, and especially during the examination phase, as well as whether participants’ self-reported SRL increases during second testing without any intervention.

Second, we were not able to validate students’ self-report measures from the pre-and posttest as well as from the learning diary by means of objective learning outcomes (e.g., exam grades). Since our sample was very heterogeneous regarding study subject and semester, exam grades were hardly comparable across students. Future studies could sample within one specific class in order to be able to compare objective learning outcomes between students with higher and lower self-reported SRL and to investigate the effect of feedback and SRL on achievement.

Third, this study has been designed to investigate the overall effects of the feedback intervention on various components of SRL. By this means, we implicitly assumed that feedback affects every learner to the same extent. However, one instructional method is rarely best for all learners (Cronbach and Snow, 1977). Hence, considering interindividual differences in the effect of feedback on daily SRL constitutes a promising direction for further research. On which days and for whom is feedback especially helpful? A within subject variation of *feed back* and *feed forward* over time would provide a first step to answer this question. Further, this would allow disentangling the effect of feedback valence and the provision of *feed forward* since, in this study, strategy suggestions were only presented if students reported low self-regulation. In the same vein, it would be interesting to investigate possible moderators of the effectiveness of daily feedback interventions as dropout analysis yielded a small trend that more self-efficacious students were more likely to cancel participation in the study. It might be that students who reported higher self-efficacy perceived daily learning diaries and feedback to be less helpful.

Fourth, based on our findings, a revision of the specific feedback sentences seems reasonable. Feedback should always include a concrete strategy recommendation in order to help students to *feed forward*. Moreover, some students reported in the qualitative evaluation at the end of the study that the feedback became boring over the course of the five-week intervention period, because formulations were similar. This might have caused some students to be less attentive or even skip the feedback. Future studies should therefore use a broad variety of feedback formulations even for one and the same construct (e.g., more than one confirmative feedback for procrastination). This might increase the subjective feeling that the feedback was really provided by a human being (as opposed to computer-generated) and that it was indeed specifically formulated for oneself.

Moreover, a drawback of the diary method is that it can be perceived as stressful for the participants to fill in a daily diary over an extended period of time, leading to increased dropout rates in empirical studies which makes it necessary to compensate the participants for their time investment (Bellhäuser et al., 2021).

Finally, although learning diaries are more proximate to the real learning behavior than trait questionnaires, one still has to keep in mind that they are a self-report measurement instruments that is not free from biases and distortions (Veenman, 2011). Thus, feedback in the present study has been generated based on self-reported study behavior and therefore relied on students’ monitoring accuracy (Nelson, 1990). Since feedback can only be effective if the learner is willing and able to actually use the feedback (Timmers and Veldkamp, 2011; van der Kleij et al., 2012), the question on how to encourage students to consider the feedback seriously constitutes another avenue for further research. Nonetheless, the present study provides a novel approach to integrate automatized, adaptive *feed back* and *feed forward* strategies in students’ daily life using a simple, parsimonious intervention.

### 4.4. Practical implication and conclusion

The results of the present study showed that automatic, individual process feedback carries the potential to foster daily SRL in an economic and cost-effective way. Process feedback that draws the learners’ attention towards the relationship between the use of a specific learning strategy and their performance helped students to improve their daily SRL.

SRL constitutes a key competence at university, but not every student knows how to self-regulate studying most effectively. In light of an increasing number of students who decide to enroll at university, offering face-to-face SRL trainings and providing individual, timely feedback to every student is hardly possible. Therefore, online learning diaries and automatic feedback offer one solution to reach a large number of students. Further, university teachers could try to implement automatized online feedback into their courses. However, irrespective of the way feedback is transmitted (orally or written, online or face-to-face), teachers should design feedback that includes all three components: *feed up*, *feed back,* and *feed forward* (Hattie and Timperley, 2007). Results showed that information on how to *feed forward* is crucial especially for students who are not satisfied with their learning outcomes. The design of effective feedback that fits situational demands and individual prerequisites of the learner constitutes a challenge for further research.

## Data availability statement

The raw data supporting the conclusions of this article will be made available by the authors, without undue reservation.

## Ethics statement

Ethical review and approval was not required for the study on human participants in accordance with the local legislation and institutional requirements. The patients/participants provided their written informed consent to participate in this study.

## Author contributions

HB originated the concept for the study and provided funding and refined the final version of the manuscript. MT and HB designed the interventions and conceptualized the study design. MT organized the data collection, performed the statistical analyses, and wrote the first draft of the manuscript. HB and CD provided feedback. All authors contributed to manuscript revision, read, and approved the submitted version.

## Funding

This project was financed by the Carl-Zeiss-Stiftung Kolleg as part of the project “Förderung des selbstregulierten Lernens in MINT-Fächern: Individualisierung eines web-basierten Trainings auf Basis von fachspezifischer und fächerübergreifender Kompetenzdiagnostik” (“*Promoting self-regulated learning in STEM subjects: Individualization of a web-based training based on subject-specific and cross-curricular competence diagnostics*”).

## Conflict of interest

The authors declare that the research was conducted in the absence of any commercial or financial relationships that could be construed as a potential conflict of interest.

## Publisher’s note

All claims expressed in this article are solely those of the authors and do not necessarily represent those of their affiliated organizations, or those of the publisher, the editors and the reviewers. Any product that may be evaluated in this article, or claim that may be made by its manufacturer, is not guaranteed or endorsed by the publisher.

## Footnotes

^1^Note that Hattie and Timperley (2007) distinguish between feedback (as a general concept) and *feed back* (as a part of effective feedback whereby learners receive information on their current performance related to their goals).

^2^Link to OSF project blinded for peer review: https://osf.io/mf58p/?view_only=711661eab32849bdb0496c740c6191a3

^3^ https://www.soscisurvey.de/
